# Adverse childhood experiences: Prevalence and related factors in adolescents of a Brazilian birth cohort^☆^

**DOI:** 10.1016/j.chiabu.2015.11.017

**Published:** 2016-01

**Authors:** Ana Luiza Gonçalves Soares, Laura D. Howe, Alicia Matijasevich, Fernando C. Wehrmeister, Ana M.B. Menezes, Helen Gonçalves

**Affiliations:** a Postgraduate Program in Epidemiology, Federal University of Pelotas, Pelotas, Brazil; b MRC Integrative Epidemiology Unit at the University of Bristol, School of Social & Community Medicine, University of Bristol, Bristol, UK; c Department of Preventive Medicine, School of Medicine, University of São Paulo (USP), São Paulo, Brazil

**Keywords:** Adverse childhood experiences, Child abuse, Risk factors, Cohort studies

## Abstract

Adverse childhood experiences (ACEs) can affect people's health and wellbeing not only at the time the ACE is experienced, but also later in life. The majority of studies on ACEs are carried out in high-income countries and little is known about its prevalence in low and middle-income countries. Thus, the aim of this study was to assess the prevalence of ACEs, associations between ACEs and sociodemographic factors, and the interrelationship between types of ACEs in adolescents of a Brazilian birth cohort. Data from 3,951 adolescents (78.4% of the original cohort) from the 1993 Pelotas Cohort were analyzed. Seven types of ACEs were assessed in those up to 18 years old: physical abuse, sexual abuse, physical neglect, emotional neglect, domestic violence, parental separation and parental death. The most common ACE was parental separation (42%), followed by emotional neglect (19.7%) and domestic violence (10.3%). Approximately 85% of the adolescents experienced at least one ACE, and females reported a higher number of adversities. Several socioeconomic, demographic and family-related characteristics were associated with the occurrence of ACEs, e.g. non-white skin color, low family income, low maternal schooling, absence of mother's partner, maternal smoking, and poor maternal mental health. A strong interrelationship was observed among the ACEs, indicating clustering of risk. These aspects should be considered by health and social care professionals in the prevention and identification of childhood adversities.

## Introduction

Adverse childhood experiences (ACEs) correspond to sources of stress that people may suffer early in life, usually before the age of 18. They are recognized as a public health problem, which can affect children's health and wellbeing not only at the time the ACE is experienced, but also later in life (Felitti et al., 1998, World Health Organization, 2015). Such experiences include multiple types of abuse (physical, sexual, and psychological), neglect, and several sorts of family dysfunction (Centers for Disease Control and Prevention, 2014, World Health Organization, 2015). In addition to having psychological consequences, various studies have shown that ACEs are associated with health-related risk factors, e.g. substance abuse and risky sexual behaviors, as well as many health outcomes such as obesity, cardiovascular diseases, cancer, and diabetes (Carr et al., 2013, Danese and Tan, 2014, Dube et al., 2003, Exley et al., 2015, Felitti et al., 1998). Furthermore, ACEs can have economic repercussions and affect social outcomes such as educational achievement and employment (Bethell et al., 2014, Liu et al., 2013).

The prevalence of ACEs varies considerably depending on the definition, the measurements, the sample characteristics, and the methodology adopted. In a meta-analysis with 217 publications that assessed the world-wide prevalence of child sexual abuse, for example, the combined prevalence was 17.7%, but it varied from 0.1% to 71%, and a significant difference was found according to the definition of sexual abuse, the source of the data (informant studies or self-report), number of questions, and geographic region, among other factors (Stoltenborgh, van Ijzendoorn, Euser, & Bakermans-Kranenburg, 2011). The occurrence of some ACEs also differs substantially according to gender, and usually females are more likely to have a higher number of childhood adversities than males (Bellis et al., 2014, Centers for Disease Control and Prevention, 2010, Cunningham et al., 2014, Dube et al., 2003, Liu et al., 2013, Stoltenborgh et al., 2011). In a study conducted by Felitti et al. (1998), whilst 8.5% of the women had 4 or more ACEs, only 3.9% of the men reported the same number of adversities before 18 years old.

Different types and combinations of risk factors have been shown to be associated with one or more ACEs (Brown et al., 1998, Thornberry et al., 2014). These determinants are present across different domains: individual, parental background, family environment, and socioeconomic context (Brown et al., 1998, Sidebotham et al., 2006, Thornberry et al., 2014). The presence of a higher number of risk factors increases the likelihood of experiencing ACEs (Brown et al., 1998, Thornberry et al., 2014). Moreover, the occurrence of multiple types of ACEs is not independent; there is clustering of events (Dong et al., 2004).

To date, the majority of studies about ACEs have been carried out in high-income countries, and more information about the prevalence of ACEs and the associated risk factors is required in low and middle-income countries, as different patterns may be found in distinct socioeconomic contexts. Thus, the aim of this study was to assess the prevalence of ACEs, the interrelationship between multiple types of ACEs, and sociodemographic correlates of ACEs in adolescents participants of a Brazilian birth cohort.

## Methods

### Socio-geographical context

When the cohort study began in 1993, Brazil was a lower-middle income country with a population of about 150 million and a gross domestic product per capita of US$ 2,710. Currently, Brazil is an upper-middle-income country with a population of 206 million, 84% of them living in the urban areas. It has a gross domestic product per capita of US$ 11,530, and the richest states are found in the Southern and South-eastern regions of the country (Instituto Brasileiro de Geografia e Estatística, 2001, The World Bank, 2015). Pelotas is a city of 342 thousands inhabitants (93% living in the urban area), located in the far south of Brazil in Rio Grande do Sul State, and with a gross domestic product per capita of US$ 8,368 (Instituto Brasileiro de Geografia e Estatística, 2012).

Brazil has a highly diverse population as a result of various processes, such as immigration (mainly Portuguese, Italian, Spanish and German), slavery (chiefly African) and considerable inter-mixing of ethnic groups. Skin color is commonly used in Brazilian surveys to categorize different ethnic groups (Instituto Brasileiro de Geografia e Estatística, 2001). According to the last census, 50.7% of the population reported black or brown skin color, and 47.7% reported themselves as having white skin color. Despite having the highest concentration of black people outside of Africa, the black population of Brazil generally occupies less qualified, poorly paid positions in the labor market, lives in areas with no or low availability of basic infrastructure services, and has more restricted access to health services (Araújo et al., 2009). Thus, skin color is usually seen as a marker of social inequality. Race and skin color distribution varies considerably across the macro-regions of the country, and in Rio Grande do Sul State, where this study was carried out, 83.2% of the population reported white skin color (Instituto Brasileiro de Geografia e Estatística, 2001).

### The sample

All mothers living in the urban area of the city of Pelotas, Southern Brazil and whose children were born alive in hospitals in 1993 (*N* = 5,249) were interviewed, and the children were followed until adolescence. More details on the methodology can be found in other publications (Araújo et al., 2010, Gonçalves et al., 2014). At the 11-year follow-up 4,452 adolescents took part in the study (87.5% follow-up rate), in the 15-year follow-up 4,325 were interviewed (85.7% follow-up rate), and in the 18-year follow-up 4,106 participated (81.3% follow-up rate) (Gonçalves et al., 2014). For this study, only people with complete data for all the adverse childhood experiences were used (*N* = 3,951; 78.4% of the original cohort).

### Measurement of adverse childhood experiences

The adverse childhood experiences assessed were: physical abuse, sexual abuse, emotional neglect, physical neglect, domestic violence, parental separation and parental death. These ACEs were selected for inclusion in our analysis based on the ACE Study (Felitti et al., 1998), although not all types of adversity measured in the ACE Study were assessed in our cohort. The questions used to evaluate the ACEs are detailed in Box 1.

Data on all ACEs except parental separation and death were obtained at the 15-year follow-up through an anonymized self-completed questionnaire. The questionnaire was handed out to the adolescents, answered in a private place, and placed in a sealed envelope after completion. Parental separation was assessed at the 15- and 18-year follow-ups, and parental death was investigated at the 11-, 15-year and 18-year follow-ups. Both parental separation and parental death were assessed through a self-reported questionnaire administered by a trained interviewer, and applied to the mothers up to 15-year follow-up and to the adolescents at the 18-year follow-up.

Socioeconomic and demographic data were collected at the perinatal, 11- and 15-year follow-ups. At the perinatal visit, the following data were collected: gender (male/female); total family income (in quintiles); mother's age (<20, 20/24, 25/29, 30/34, ≥35); number of siblings (0, 1, 2 and 3 or more); maternal schooling (0–4, 5–8, 9–11, and 12 or more years); presence of mother's partner living in the house (yes/no), maternal smoking during pregnancy (yes/no), and maternal alcohol use during pregnancy (yes/no). At the 11-year follow-up, the following data were collected: skin color (white, black, brown, yellow, indigenous), maternal smoking (never smoker, former smoker, current smoker), maternal daily intake of alcohol (yes/no), maternal mental health, measured using the short-version of the Self-Reporting Questionnaire–SRQ-20, and presence of at least one unemployed household member (yes/no). Women were divided into two groups for the SRQ-20: no common mental disorder (a score less than or equal to seven) and common mental disorder (a score equal to or greater than eight) (Mari & Williams, 1986). At the 15-year follow-up the following data were collected: family income (continuous) and presence of at least one unemployed household member (yes/no). To assess the family income change between birth and 15 years old, a variable was generated by using tertiles of family income in both periods. The participants were classified in the following categories: always poor (those belonging to the lowest tertile of income at both in birth and 11 years); poor → non-poor (lowest tertile to middle or highest tertile); non-poor → poor (middle or highest tertile to lowest tertile); and never poor (middle or highest tertile at both time points).

### Statistical analysis

Initially, each ACE was evaluated separately. Subsequently an ACE score was generated, where each affirmative answer was worth one point, such that the total score could vary from 0 (no ACE) to 7 points (exposed to all adverse events). The prevalence of each ACE was assessed, and the distribution of the score was examined using a histogram. The ACE score was then categorized into 0, 1, 2, 3, and 4 or more adversities due to low prevalence of more than 4 ACEs. Multinomial logistic regression was used to calculate the odds ratios (OR) and their respective 95% confidence intervals (95% CI) for the associations between potential risk factors and the number of ACEs, and to calculate the OR of the interrelationships between types of ACEs. For associations between potential risk factors and the number of ACEs, we present associations adjusted for potential confounders; the choice of confounders differs between the potential risk factors (e.g. there are no potential confounders for skin color, as all other variables would be expected to be on the causal pathway from skin color to ACEs). The significance level adopted was 5%. Effect modification by gender was tested and no evidence for interaction was found.

The analyses were performed in the software Stata 13.0^®^ (Statcorp, College Station, TX, USA).

### Ethical approval

This study was approved by the Research Ethics Committee of the Medical School of the Federal University of Pelotas under protocol 40600026. After agreeing to take part in the study, the mothers (or caregivers) and adolescents provided written informed consent.

## Results

Table 1 shows the socioeconomic and demographic characteristics of the participants included in the analysis and the comparison with participants with missing data or those lost to follow-up. Amongst included participants, approximately 52% of the adolescents were female, and the majority of them reported white skin color. Roughly 45% of the adolescents were never poor from birth to 15 years old, and about 21% of them were always poor. Almost 50% of the mothers have studied between 5 and 8 years and nearly 30% of them were between 20 and 24 years old when the adolescent was born. For 41% of the mothers, the cohort participant was the first child, and 11.5% of them were not living with a partner when the adolescent was born. One third of the mothers smoked during pregnancy, and 5.3% of them consumed any alcohol beverage. Common mental disorders were present in 40% of the mothers, and 23.4% of the adolescents had at least one household member who was unemployed when they were both 11 and 15 years old. We observed some differences between participants included in our analysis and those excluded due to missing data or loss to follow-up, e.g. those excluded were more likely to be male, to have more siblings, to have a mother living without a partner, and to have a mother with common mental health disorders.

The prevalence of ACEs is presented in Table 2. Almost 7% of the adolescents reported experiencing physical abuse, which was more frequent among females (8.3%) than males (5.4%). Sexual abuse was reported by 1.4% of the participants, and this was also more commonly reported by females (2.2%) than males (0.5%). About 20% of the adolescents reported having experienced emotional neglect; the prevalence of this was 2.2 times higher in females than males. Physical neglect was reported by 4.6% of the adolescents, with similar prevalence among males and females, and 12.7% of the female adolescents have experienced domestic violence, whereas 7.8% of the males reported this experience. Parental separation was reported by 42% of the adolescents, and parental death by 10.1% of them, both similar across males and females. Father's death (8.3%) was more frequent than mother's death (2.2%).

**Fig. 1:**
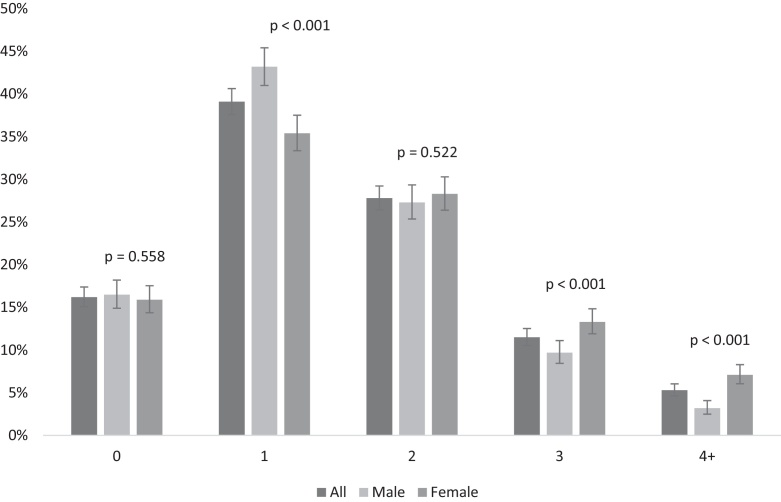
Score of the number of adverse childhood experiences, according to gender. 1993 Pelotas Birth Cohort, Brazil (*n* = 3,951).

Fig. 1 shows the score of the number of adverse childhood experiences reported by males and females. About 85% of the adolescents have experienced at least one adverse event during childhood, and female adolescents tended to report a greater number of ACEs than males. The occurrence of three ACEs was reported by 13.3% of the females and 9.7% of the males. Furthermore, the occurrence of four or more ACEs was reported by 7.1% of the females and 3.2% of the males.

Table 3 shows the pairwise associations between each of the ACEs adjusted for gender, skin color, family income at birth, and mother's schooling. There were positive associations between almost all pairs of ACEs, and the strongest associations were seen for those who have experienced physical abuse, sexual abuse, emotional neglect, and domestic violence. Adolescents who experienced physical abuse had odds 9.6 times higher of experiencing domestic violence and vice versa. Those adolescents who experienced emotional neglect had odds 9.1 and 8.2 times higher of experiencing sexual abuse and physical abuse, respectively. A negative association was observed between parental separation and parental death, and parental death was the only ACE which had no association with the other ACEs other than parental separation.

Table 4 presents the adjusted odds ratios for the number of ACEs according to socioeconomic, demographic and family-related characteristics. Adolescents with non-white skin color had greater OR of ACEs; the odds of experiencing 4 or more childhood adversities among non-white adolescents was 2.3 times the odds among white adolescents. The likelihood of having a greater number of ACEs was higher in those adolescents in the lowest quintile of family income compared to those in the highest quintile. Change in family income between birth and age 15 also showed some association with ACEs, which was stronger for those who were not poor at birth and became poor at 15 years old. An inverse relationship was observed for maternal schooling and mother's age with the number of ACEs experienced; the lower the mother's education and the mother's age at the time of the adolescent's birth, the higher the number of ACEs. There was no association between number of siblings and the occurrence of ACEs. Adolescents whose mothers had no partner when they were born had a higher number of ACEs, as well as those whose mothers smoked and drank alcohol during pregnancy. A positive association was observed for maternal mental health problems and presence of at least one unemployed household member with the number of ACEs.

## Discussion

This study described the prevalence of ACEs and its related socioeconomic, demographic and family-related factors in adolescents of a Brazilian birth cohort. The most common ACE observed was parental separation, followed by emotional neglect and domestic violence. A strong interrelationship was observed among the ACEs, especially between domestic violence and physical abuse, and between emotional neglect and sexual abuse. Female adolescents were more likely to have a higher score of ACEs, and the number of ACEs was also related to socioeconomic and demographic characteristics, as well as maternal smoking, maternal mental health, and presence of an unemployed household member.

### Prevalence of ACE

More than 80% of the adolescents in this cohort have experienced at least one ACE. This prevalence of one or more ACE is higher than was found in other studies carried out in developed countries (Bellis et al., 2014, Bethell et al., 2014, Cunningham et al., 2014, Dube et al., 2003), mainly due to the very high prevalence of parental separation.

The prevalence of sexual abuse, physical abuse, physical neglect, and domestic violence observed in this study were lower than in other studies carried out either in Brazil or in other countries, even using similar questions to assess these adversities (Barbosa et al., 2014, Bellis et al., 2014, Cunningham et al., 2014, Dube et al., 2003, Li et al., 2014, Logan-Greene et al., 2014, Polanczyk et al., 2003). It is possible that some of these events happen later in adolescence and therefore these ACEs have not been captured in their entirety in this study, as the majority of the ACEs were evaluated in adolescents at 15 years of age. However, evidence from other studies suggests this is unlikely to have biased our results as the ACEs are more likely to occur in young infants or soon after the onset of puberty (Krug, Dahlberg, Mercy, Zwi, & Lozano, 2009). Another possibility is that younger people, chiefly younger adolescents, could feel uncomfortable reporting the occurrence of these events, even using a confidential questionnaire, thus underestimating the prevalence. It is also possible that younger people do not perceive some experiences as adversities; in some studies higher prevalence of sexual abuse, physical abuse and domestic violence were found among older people, potentially due to age differences in perception of experiences (Bellis et al., 2014, Polanczyk et al., 2003). It is also possible that some ACEs might not have been captured fully by the questions used in this study (e.g. touching and fondling may have been missed by the sexual abuse question, the physical neglect question might represent poverty as well as/instead of neglect, and the domestic violence question may have only captured physical violence but not other forms of violence), thus underestimating the prevalence of these ACEs.

The prevalence of emotional neglect was similar to other studies (19% compared with approximately 15% in previous studies (Barbosa et al., 2014, Dube et al., 2003)). The slightly higher prevalence of emotional neglect might be explained by the questions used to identify this ACE, which were different from other studies that reported lower prevalence.

On the other hand, the prevalence of parental separation was almost twice the prevalence found in some studies performed in England and the United States (42% compared to circa 22%) (Bellis et al., 2014, Bethell et al., 2014, Cunningham et al., 2014), but lower than another study carried out in Brazil with low-income schoolchildren, which found 53.4% parental divorce (Avanci, Assis, Oliveira, & Pires, 2012). Parental death (10.1%) also had a higher prevalence than in other studies conducted in developed countries, which varied from 2.6% to 7.4% (Bethell et al., 2014, Li et al., 2014, Stikkelbroek et al., 2012). However, it was lower than in a multi-country study, which found 17% early parental death (before the age of 16) and 25% in Brazil (Phillips & Carver, 2015), although it was evaluated in a poorer region of Brazil than this study. The prevalence of parental death in middle-income countries is higher than in high-income countries (Phillips & Carver, 2015), and other studies also show that father's death is more common than mother's death (Li et al., 2012). The loss of a parent is always a difficult situation to deal with, especially if it occurs early in life. Similar to parental separation, parental death requires a number of adjustments within the family, which can be very stressful. The cause of death was not assessed in this study, and the impact on a child whose parent dies suddenly might be very different than for a child whose parent's death is expected.

### Interrelationships between ACEs

An interrelationship among almost all the ACEs was observed in this study, indicating a clustering of risk. For some types of ACEs, the relationships were very strong with an OR of approximately 10. Nevertheless, the strong relationship between some ACEs (in particular physical abuse and domestic violence) could reflect the wording used to assess these ACEs, with some participants potentially answering both questions referring to the same incident.

Interrelatedness of multiple forms of childhood abuse, neglect, and household dysfunction has been reported by other authors (Dong et al., 2004, Dube et al., 2003, Felitti et al., 1998). This strong clustering of ACEs suggests that the adversities cannot and should not be regarded as independent events when examining prevalence, risk factors and consequences. There is evidence that different forms of ACEs share some risk factors, and that there is no single cause of ACEs, but multiple and interacting factors at different levels: individual, parental, familial and social (Brown et al., 1998, Dong et al., 2004, Thornberry et al., 2014).

### Potential risk factors for ACE

A greater occurrence of ACEs in female, non-white, less educated and poorer people observed in this study has also been observed in other studies (Bellis et al., 2014, Brown et al., 1998, Logan-Greene et al., 2014, Sidebotham et al., 2006, Ye and Reyes-Salvail, 2014). In our study, the income change from birth to adolescence also seems to play a role in the prevalence of ACEs; the odds ratio of ACEs was highest in those who were not poor at birth and became poor at 15 years old. The worsening in the economic situation could be related to parental stress, which may make children more vulnerable to ACEs occurrence (Stith et al., 2009). However, it is important to interpret this association with caution, since the family income change could be a consequence of some of the adversities, particularly parental separation (or parental abandonment in some cases) or parental death.

The occurrence of ACEs was inversely related to mother's age at delivery, corroborating findings from previous studies (Brown et al., 1998, Sidebotham et al., 2001, Sidebotham et al., 2006, Stith et al., 2009). This association could reflect socioeconomic position, as more educated women are more likely to delay childbearing (Balasch & Gratacos, 2012). However, this association remained after adjusted for mother's education and family income. Older women are more likely to have planned their pregnancies and may have better parenting abilities than younger mothers, as well as more experience and knowledge (Sidebotham et al., 2001), and this could result in a lower probability of ACEs in their children.

The absence of a mother's partner when the adolescent was born increased the odds of ACEs, but the number of siblings was not associated with childhood adversities. Other studies found family size and single parenthood to be risk factors for child maltreatment (Sidebotham et al., 2001, Sidebotham et al., 2006, Stith et al., 2009). This association could reflect either a lack of resources – economic, physical or psychological – to meet the child's needs, or a lack of awareness or appreciation of the child's needs (Sidebotham et al., 2001).

A higher odds ratio of ACEs was observed in those adolescents whose mothers smoked during pregnancy. Although prenatal smoking is closely related to maternal education and income (Cnattingius, 2004), the association remained after adjustment for mother's schooling and family income. Smoking during pregnancy has been related to impaired childrearing behaviors and family problems (Cnattingius, 2004), which could be related to the higher risk of ACEs. Some studies have shown that stress enhances vulnerability to tobacco use in females, and smoke is commonly used as a mechanism of coping with anxiety and stress (Torres & O’Dell, 2015). Thus, the maternal smoking could be associated to a stressful family environment, which could play a role in the occurrence of ACEs.

In our study, maternal alcohol intake during pregnancy and maternal mental health problems were associated with a higher occurrence of ACEs. Some studies have shown parental psychiatric history, including alcohol abuse and psychiatric illness, as a risk factor for child maltreatment (Brown et al., 1998, Sidebotham et al., 2006, Stith et al., 2009). In this study we could not determine if mothers abused alcohol during their pregnancy because only limited information about their alcohol use (whether she drank at all or not) was available. As with smoking, alcohol is a commonly used mechanism to cope with stress. Thus, maternal alcohol intake during pregnancy could reflect a problematic environment, which could be related to several adversities. In addition, people with psychiatric disorders are more likely to use alcohol, and alcohol intake during pregnancy could be associated with maternal mental health problems. The associations between maternal common mental disorders and ACEs have to be interpreted carefully though, as the ACEs could have occurred before 11 years old, when maternal mental health was evaluated.

The presence of an unemployed household member was associated with a higher odds ratio of ACEs. Parental unemployment has been related to child neglect (Stith et al., 2009) and maternal employment was found to have a protective effect in the occurrence of child maltreatment (Sidebotham et al., 2006). The benefits could be through the impact of the job on parental stress and self-esteem, as well as direct effects on the mother–child relationship (Sidebotham et al., 2006). Additionally, unemployment could play a role in the risk of ACEs through economic factors. In this study we did not assess which household member was unemployed and how long he/she had been unemployed, and it is therefore difficult to compare with other studies. The temporality of this association has to be considered as well.

### Strengths and limitations of this study

The data on ACEs are self-reported, which could potentially introduce reporting bias. However, there is no gold standard for collecting data on ACEs – ‘objective’ data from official records are also likely to be biased by under-reporting (Stoltenborgh et al., 2011). The data in this study were collected during adolescence, and as such the potential for under-reporting may be less than in studies where ACEs are reported during adulthood. On the other hand, the occurrence of ACEs could be under-reported in adolescents compared with adults if younger people feel less confident to report ACEs, or if they do not yet have the context to understand their experience of ACE and classify it as such.

An important limitation is that the timing of the ACEs was not ascertained, and some of the potential risk factors we have examined may have occurred after the ACEs, meaning reverse causality is possible. However, the aim of this study was to assess factors related to ACEs rather than to provide evidence of causal relationships.

Despite the high retention rate in this cohort, we do have some missing data, and missingness was associated with some characteristics likely to be associated with ACEs, e.g. maternal common mental disorder. It is therefore possible that the prevalence of ACEs identified in this analysis is underestimated, but the associations between risk factors and ACEs are less likely to be affected by this missing data (Nohr, Frydenberg, Henriksen, & Olsen, 2006).

This study described the prevalence of ACEs and its related factors in a middle-income country, using prospective data from a birth cohort with a high follow-up rate. The assessment of these adversities in a different socioeconomic and demographic context is important as the majority of the studies are carried out in high-income countries, and different prevalence and risk factors may be expected in different contexts. Our study used questions to determine the occurrence of ACEs that were similar to those used in other studies, facilitating comparisons. Unfortunately, several other ACEs of interest (e.g. emotional abuse, household substance abuse, household mental illness, death of a sibling, etc.) were not assessed in this study. Other studies should be conducted in low- and middle-income countries to assess a wider range of ACEs.

In conclusion, this study showed a greater prevalence of at least one ACE than in studies in high-income settings, chiefly due to a high rate of parental separation. We observed strong interrelationships among the ACEs, highlighting that these events do not occur independently. We found a higher occurrence of child adversities in females compared with males. Several socioeconomic and demographic risk factors are related to the occurrence of ACEs, consistent with previous studies in other contexts.

## Conflicts of interest

The authors declare no known conflicts of interest associated with this publication.

## Figures and Tables

**Table 1:** Socioeconomic, demographic, and family-related characteristics of participants with complete data compared with participants with missing data or lost to follow-up. 1993 Pelotas Birth Cohort, Brazil.

Variables	Participants included in the analysis %	Participants excluded from the analysis %	*p* value¥
**Gender**	*N* = 3,951	*N* = 1,297	0.001
Male	48.3	53.7	
Female	51.7	46.3	
**Skin color**	*N* = 3,951	*N* = 469	0.369
White	66.6	68.7	
Non-white	33.4	31.3	
**Family income at birth (quintile)**	*N* = 3,951	*N* = 1,185	0.017
1st (lowest)	19.2	22.9	
2nd	23.6	22.2	
3rd	17.5	16.8	
4th	20.2	17.2	
5th (highest)	19.6	20.9	
**Income change from 0 to 15 years**	*N* = 3,898	*N* = 298	0.656
Always poor	21.4	23.8	
Non poor–poor	11.5	12.4	
Poor–non poor	21.5	21.5	
Never poor	45.6	42.3	
**Mother's schooling (years)**	*N* = 3,951	*N* = 1,291	<0.001
0–4	26.7	31.8	
5–8	48.4	39.7	
9–11	17.3	18.6	
12 or more	7.6	9.8	
**Mother's age at birth (years)**	*N* = 3,951	*N* = 1,291	0.126
<20	17.0	18.8	
20–24	27.5	27.9	
25–29	25.5	26.5	
30–34	19.0	16.0	
≥35	11.0	10.8	
**Number of siblings**	*N* = 3,951	*N* = 1,291	0.049
0	40.8	39.8	
1	30.2	27.7	
2	15.7	16.6	
3 or more	13.3	15.9	
**Mother living with partner**	*N* = 3,951	*N* = 1,298	<0.001
Yes	88.5	84.9	
No	11.5	15.1	
**Maternal smoking during pregnancy**	*N* = 3,951	*N* = 1,298	0.799
No	66.7	66.3	
Yes	33.3	33.7	
**Mother consumed any alcohol during pregnancy**	*N* = 3,951	*N* = 1,298	0.189
No	94.7	95.6	
Yes	5.3	4.4	
**Mother's mental health (SRQ-20)**	*N* = 3,951	*N* = 1,298	<0.001
No common mental disorders	60.0	20.3	
Common mental disorders	40.0	79.7	
**At least one household member unemployed at 11 years and 15 years**	*N* = 3,947	*N* = 464	0.499
No	76.6	78.0	
Yes	23.4	22.0	

¥ Chi-square test for the difference between participants with complete data and participants with missing data or lost to follow-up.

**Table 2:** Prevalence of adverse childhood experiences, according to gender. 1993 Pelotas Birth Cohort, Brazil (*N* = 3,951).

Variables	All (*N* = 3,951) % (CI 95%)	Male (*N* = 1,909) % (CI 95%)	Female (*N* = 2,042) % (CI 95%)	*p* value¥
Physical abuse	6.9 (6.1, 7.7)	5.4 (4.5, 6.6)	8.3 (7.2, 9.5)	0.001
Sexual abuse	1.4 (1.1, 1.8)	0.5 (0.3, 1.0)	2.2 (1.7, 3.0)	<0.001
Emotional neglect	19.7 (18.5, 21.0)	12.2 (10.8, 13.7)	26.8 (24.9, 28.7)	<0.001
Physical neglect	4.6 (4.0, 5.3)	5.2 (4.3, 6.3)	4.1 (3.3, 5.1)	0.112
Domestic violence	10.3 (9.4, 11.3)	7.8 (6.7, 9.1)	12.7 (11.3, 14.2)	<0.001
Parental separation	42.0 (40.4, 43.5)	40.9 (38.8, 43.1)	42.9 (40.8, 45.1)	0.197
Parental death	10.1 (9.2, 11.1)	9.6 (8.4, 11.0)	10.6 (9.3, 12.0)	0.342
Mother's death	2.2 (1.8, 2.3)	2.0 (1.5, 2.8)	2.4 (1.9, 3.2)	0.453
Father's death	8.3 (7.4, 9.2)	8.0 (6.7, 9.3)	8.6 (7.4, 9.9)	0.525

¥ Fisher's exact test for difference in prevalence by gender.

**Table 3:** Adjusted odds ratios (OR) and 95% confidence interval for pairwise associations between adverse childhood experiences. 1993 Pelotas Birth Cohort. Pelotas, RS, Brazil (*N* = 3,951).

	Odds ratio for additional ACE^a^						
Experienced event:	Physical abuse	Sexual abuse	Emotional neglect	Physical neglect	Domestic violence	Parental separation	Parental death
	OR (95% CI)	OR (95% CI)	OR (95% CI)	OR (95% CI)	OR (95% CI)	OR (95% CI)	OR (95% CI)
Physical abuse		4.1 (2.2, 7.7)*	8.2 (6.2, 10.7)*	4.9 (3.3, 7.1)*	9.6 (7.3, 12.6)*	1.4 (1.1, 1.8)**	1.1 (0.8, 1.6)
Sexual abuse	4.1 (2.2, 7.7)*		9.0 (4.9, 16.6)**	6.7 (3.5, 12.9)**	2.5 (1.3, 4.6)**	2.1 (1.2, 3.7)**	1.3 (0.6, 2.7)
Emotional neglect	8.2 (6.2, 10.7)*	9.1 (4.9, 16.7)*		5.2 (3.8, 7.2)*	5.1 (4.1, 6.4)*	1.6 (1.3, 1.8)*	0.9 (0.7, 1.2)
Physical neglect	4.8 (3.3, 7.0)*	7.2 (3.7, 13.8)*	5.1 (3.7, 7.0)*		3.6 (2.5, 5.1)*	2.4 (1.8, 3.4)*	1.4 (0.9, 2.2)
Domestic violence	9.6 (7.3, 12.6)*	2.5 (1.3, 4.6)**	5.1 (4.1, 6.4)*	3.6 (2.5, 5.1)*		2.2 (1.8, 2.8)*	1.1 (0.8, 1.5)
Parental separation	1.4 (1.1, 1.8)**	2.1 (1.2, 3.7)**	1.6 (1.3, 1.8)*	2.4 (1.8, 3.4)*	2.2 (1.8, 2.8)*		0.7 (0.6, 0.9)**
Parental death	1.1 (0.7, 1.6)	1.3 (0.6, 2.7)	0.9 (0.7, 1.2)	1.4 (0.9, 2.2)	1.1 (0.8, 1.5)	0.7 (0.6, 0.9)**	

Adjusted for gender, skin color, family income at birth, and mother's schooling.

a For example: Among persons who were physically abused, the odds of experiencing sexual abuse was 4.1 times higher than those who were not physically abused.

* *p* value < 0.001.

** *p* value < 0.05.

**Table 4:** Adjusted odds ratio of ACE score according to socioeconomic, demographic, and family-related characteristics, estimated by multinomial logistic regression. 1993 Pelotas Birth Cohort, Brazil (*N* = 3,951).

Variables	Number of ACEs				
	0	1	2	3	4+
**Skin color**					
White	1	1	1	1	1
Non-white	1	1.3 (1.1, 1.7)*	2.1 (1.7, 2.6)*	2.0 (1.5, 2.6)*	3.1 (2.2, 4.3)*
**Family income at birth (quintile)**^a^					
1st (lowest)	1	1.3 (1.0, 1.7)	1.8 (1.3, 2.4)**	2.7 (1.8, 3.9)*	2.4 (1.4, 3.9)**
2nd	1	1.2 (0.9, 1.7)	1.6 (1.2, 2.3)*	2.3 (1.5, 3.6)*	1.6 (0.9, 2.8)
3rd	1	1.2 (0.9, 1.6)	1.3 (0.9, 1.7)	2.1 (1.4, 3.2)*	1.6 (0.9, 2.8)
4th	1	1.2 (0.9, 1.6)	1.2 (0.9, 1.7)	1.7 (1.2, 2.6)*	1.5 (0.9, 2.6)
5th (highest)	1	1	1	1	1
**Income change from 0 to 15 years**^a^					
Always poor	1	1.5 (1.2, 2.0)**	2.2 (1.6, 2.9)*	3.1 (2.3, 4.4)*	3.5 (2.3, 5.4)*
Non poor–poor	1	2.2 (1.5, 3.2)*	2.6 (1.8, 3.9)*	4.6 (3.0. 7.1)*	6.1 (3.6, 10.3)*
Poor–non poor	1	1.1 (0.9, 1.4)	1.5 (1.2, 1.9)**	1.6 (1.1, 2.2)**	1.6 (1.0, 2.5)**
Never poor	1	1	1	1	1
**Mother's schooling (years)**^a^					
0–4	1	1.9 (1.4, 2.7)*	2.8 (1.9, 4.1)*	3.2 (1.9, 5.4)*	6.1 (2.5, 14.8)*
5–8	1	1.9 (1.4, 2.6)*	2.4 (1.7, 3.5)*	2.7 (1.7, 4.5)*	4.1 (1.8, 9.9)**
9–11	1	1.6 (1.1, 2.2)**	1.7 (1.1, 2.5)**	1.5 (0.8, 2.5)	2.6 (1.0, 6.7)**
12 or more	1	1	1	1	1
**Mother's age at birth (years)**^b^					
<20	1	1.8 (1.2, 2.7)**	2.4 (1.6, 3.6)*	2.7 (1.6, 4.4)*	3.6 (1.8, 7.0)*
20–24	1	1.3 (0.9, 1.7)	1.4 (1.0, 1.9)	1.5 (0.9, 2.3)	2.1 (1.1, 3.9)**
25–29	1	1.3 (0.9, 1.8)	1.2 (0.9, 1.7)	1.4 (0.9, 2.1)	1.8 (1.0, 3.5)
30–34	1	1.1 (0.8, 1.5)	1.0 (0.7, 1.4)	1.1 (0.7, 1.8)	1.2 (0.6, 2.4)
≥35	1	1	1	1	1
**Number of siblings**^b^					
0	1	1	1	1	1
1	1	0.8 (0.6, 1.0)	0.8 (0.6, 1.0)	0.7 (0.5, 0.9)**	1.0 (0.7, 1.4)
2	1	0.9 (0.6, 1.1)	0.9 (0.7, 1.3)	0.8 (0.6, 1.2)	1.3 (0.9, 2.1)
3 or more	1	0.9 (0.7, 1.2)	0.9 (0.6, 1.2)	0.9 (0.6, 1.4)	0.9 (0.5, 1.5)
**Mother living with partner**^b^					
Yes	1	1	1	1	1
No	1	3.1 (1.9, 5.1)*	5.8 (3.5, 9.6)*	6.9 (4.0, 11.7)*	8.6 (4.8, 15.3)*
**Mother consumed any alcohol during pregnancy**^b^					
No	1	1	1	1	1
Yes	1	1.8 (1.1, 3.0)**	1.4 (0.8, 2.4)	2.1 (1.2, 3.7)**	2.7 (1.4, 5.3)**
**Maternal smoking during pregnancy**^b^					
No	1	1	1	1	1
Yes	1	1.0 (0.8, 1.2)	1.4 (1.1, 1.8)**	1.6 (1.2, 2.1)**	1.9 (1.4, 2.7)*
**Mother's mental health (SRQ-20)**^b^					
No common mental disorder	1	1	1	1	1
Common mental disorders	1	1.5 (1.2, 1.8)*	1.8 (1.5, 2.3)*	2.5 (1.9, 3.3)*	3.5 (2.5, 5.0)*
**At least one household member unemployed at 11 and 15 years**^b^					
No	1	1	1	1	1
Yes	1	1.2 (0.9, 1.5)	1.5 (1.1, 1.9)**	1.5 (1.1, 2.0)**	2.1 (1.4, 3.0)*

a Adjusted for gender and skin color.

b Adjusted for gender, skin color, family income at birth, and mother's schooling.

* *p* value < 0.001.

** *p* value < 0.05.

## Box 1 Questions used to assess adverse childhood experiences

**Table:**

Physical abuse:	Has an adult of your family or someone who was looking after you hit you in a way that left you hurt or bruised?
Sexual abuse:	Has anyone ever tried to do sexual things to you against your will, threatening or hurting you?
Emotional neglect:	Have you ever thought or felt that your parents did not want you to have been born?
or
Have you ever thought or felt that someone in your family hates you?
Physical neglect:	Have you ever not had enough food at home or had to wear dirty or worn clothes because you had no others?
Domestic violence:	Has there ever been fights with physical assault in your household between adults or has an adult ever assaulted a child or adolescent?
Parental separation:	Did you and <name>’s father separate?
or
Are your parents separated?
Parental death:	Is <name>’s natural mother/father still alive?
or
Are your mother/father still alive or has she/he died?
